# Analysis of shared underlying mechanism in neurodegenerative disease

**DOI:** 10.3389/fnagi.2022.1006089

**Published:** 2022-11-29

**Authors:** Rickeem Butler, David Bradford, Kathleen E. Rodgers

**Affiliations:** Department of Medical Pharmacology, Center for Innovation in Brain Science, University of Arizona College of Medicine, Tucson, AZ, United States

**Keywords:** Alzheimer’s disease, amyotrophic lateral sclerosis, multiple sclerosis, bioenergetics, inflammation, immune, metabolic

## Abstract

In this review, the relationship between bioenergetics, mitochondrial dysfunction, and inflammation will be and how they contribute to neurodegeneration, specifically in Alzheimer’s disease (AD), amyotrophic lateral sclerosis (ALS), and multiple sclerosis (MS) will be reviewed. Long-term changes in mitochondrial function, autophagy dysfunction, and immune activation are commonalities shared across these age-related disorders. Genetic risk factors for these diseases support an autophagy-immune connection in the underlying pathophysiology. Critical areas of deeper evaluation in these bioenergetic processes may lead to potential therapeutics with efficacy across multiple neurodegenerative diseases.

## Disease background and pathology development

### Alzheimer’s disease

Of the dementias, AD is the most common disorder affecting the elderly to date. In fact, a comprehensive study looking at the global, regional, and national burden of diseases and injuries for adults 70 years and older, reported that AD was consistently in the top five causes of death in 51 countries (GBD 2019 Ageing Collaborators, 2022). AD currently affects approximately 50 million people worldwide (Alzheimer’s Disease International, 2018) and is expected to increase to at least 150 million by 2050; incurring nearly $1.1 trillion in medical care costs to the United States economy alone (2020 Alzheimer’s Association Report, 2020).

Alzheimer’s disease is a progressive neurodegenerative disorder that can manifest in a continuous decline in cognitive function, which gradually destroys a person’s memory and ability to learn, reason, communicate, and carry out daily activities (Rubio-Perez and Morillas-Ruiz, 2012). Researchers agree that there are two major neuropathologic hallmarks that underscore the disease’s progression: the development of neurofibrillary tangles due to hyperphosphorylation of tau, and neuritic plaques due to aggregation of improperly cleaved amyloid beta (Aβ). In concert, this hinders cell sustaining connectivity and nutritional transport and resulting in neurodegeneration and cell death (Iqbal et al., 2010; Cao et al., 2012).

*APOE4* is a significant risk factor for the development of AD and is a driving factor of cognitive decline in healthy perimenopausal women with a poor metabolic profile. Numerous studies show that *APOE4* is also a risk factor for other diseases, including cerebral amyloid angiopathy, dementia with Lewy bodies, tauopathy, cerebrovascular disease, multiple sclerosis, and vascular dementia, as well as being related to poor outcome following head injury. This may be in part due to the increased inflammatory responses upon exposure to *APOE4*. Evidence for the contribution of APOE4 to the pathogenesis of AD stems from familial studies. To date, prospective studies on healthy aging and longevity have established the APOE ɛ4 allele as a risk factor for poorer episodic memory in older adults (van Duijn et al., 1994; Seripa et al., 2011; Wilson et al., 2011). Members carrying at least one copy of the ɛ4 allele had lower scores in both immediate and delayed recall compared to non-ɛ4 allele carriers (Du et al., 2021).

Despite the identification of these disease markers, there is no fully efficacious treatment for AD aimed at neuroprotection and recovery (Pais et al., 2020). Current therapies prioritize symptom management and while some have been proven to slow disease progression (Marucci et al., 2021), none yet convincingly alters disease pathology. This is due to AD’s branched and varied pathology that in turn has spawned branched and varied hypotheses to attempt to understand etiologies. However, given their far-reaching systemic effects and their association with aging, there is a growing consensus that a sustained inflammatory environment and altered oxidative metabolism underpin AD pathology (Rubio-Perez and Morillas-Ruiz, 2012; Swerdlow, 2012).

### Amyotrophic lateral sclerosis

Amyotrophic lateral sclerosis, commonly known as Lou Gehrig’s Disease, is a devastating, fatal neurodegenerative disease characterized by a progressive loss of both the upper motor neuron (UMN)s and lower motor neurons (LMN; Rowland and Shneider, 2001). This loss of both upper and lower motor neurons makes ALS unique from the other motor neuron diseases, which typically affects one or the other. ALS is classified as an orphan disease, affecting around 1/100,000 people (Zarei et al., 2015). First diagnosed in 1869 (Magnussen and Glass, 2017; Ma and Li, 2019), ALS was believed to only affect motor neurons, but sentiment has shifted toward a multisystem neurodegenerative disorder, also affecting behavior and cognition (van Es et al., 2017). To date, there are only two FDA-approved treatments—Riluzole, Edaravone, and Relyvrio (the latter with provisional approval), which provide a limited therapeutic benefit (Mayo Clinic, 2022).

ALS has been challenging to research and treat due to the heterogeneity of the disease. About 90% of all cases are sporadic (sALS), having no obvious genetic component. The remaining 10% are familial (fALS), having an inherited genetic component (Chen et al., 2013; Kirby et al., 2016). There are over 50 genes associated with the development of ALS, with the 4 main genes associated being C9orf72, SOD1, TARDBP, and FUS (Mejzini et al., 2019). Mutations in TDP-43 and FUS are associated with increased risk of developing ALS. M TDP-43 mutations and aggregates have also been found in patients with frontotemporal dementia and AD (Nag et al., 2018; Sahoo et al., 2018). A study evaluating genetic risks shared between ALS and AD found two additional possible causal variants in GAK and TSPOAP1-AS1 genes (van Rheenen et al., 2021). While the exact mechanism of disease onset and progression remains elusive, it is believed that motor neuron death results from a combination of mitochondrial dysfunction, glutamate excitotoxicity, oxidative stress, neuroinflammation, and the formation of protein aggregates (Bonafede and Mariotti, 2017).

### Multiple sclerosis

Multiple sclerosis (MS) is a debilitating disease that arises from demyelination of axons due to chronic inflammatory processes in the central nervous system (CNS). Most recent estimates indicate that the global prevalence of MS is 35.9 per 100,000 with a higher incidence in the Americas, 117.5 per 100,000 (Walton et al., 2020). Currently, it is believed that T cells lead the demyelination events, although antibodies and vulnerability of myelin, oligodendrocytes, and axons are also involved in the disease process. This diversity contributes to the heterogeneous phenotypes of patients diagnosed with MS. Demyelination and axonal loss contributed to the formation of white-matter lesions that are the hallmark of MS. Eventually, these plaques become chronic with limited demyelination with preservation of axons and the formation of an astrocytic scar. The composition of the typical periventricular white-matter lesions is heterogeneous and may point to the various pathophysiological processes that lead to the formation of these lesions. The lesions appear to originate from within small venules in this area of the brain. Fresh lesions or plaques are highly inflammatory and show an abundance of monocytes, activated microglia, T lymphocytes from different phenotypes, and B cells (Metz et al., 2014). Demyelination, various degrees of axonal damage, and, at later stages, glial scarring are all hallmarks of MS plaques. The symptoms associated with the development of MS is, in part, dependent upon the site of the lesions. There are 4 forms of MS, clinically isolated syndrome [one clinical event (CIS)], relapsing remitting (RRMS; 85% of patients are initially diagnosed), primary progressive (PPMS), and secondary progressive (SPMS, National Multiple Sclerosis Society, 2022). Initiating events for the development of MS lesions include immune activation/inflammation and mitochondria dysfunction.

Evidence for the contribution of genetic factors to the pathogenesis of MS stems from family and twin studies (Doolittle et al., 1990; McFarland, 1992; Sadovnick et al., 1993; Ebers and Sadovnick, 1994). As with other autoimmune diseases, immune genes and immune response genes are among the top genetic polymorphisms that contribute to increase MS risk (Muñoz-Culla et al., 2013). To date, population studies have demonstrated an association, in Caucasian MS patients, with the class II major histocompatibility complex (MHC) alleles DRB1*1501, DRB5*0101, and DQB1*0602 (Vartdal et al., 1989; Oksenberg and Steinman, 1990; Ebers et al., 1996). These alleles are all contained in the DR2 haplotype, the one that has been most consistently associated with the disease. Further, HLADRB1*1501 has been associated with markers of disease severity (Isobe et al., 2016). HLA-DRB1*1501 was also identified as genetic risk factor for the development of late-onset AD (Steele et al., 2017; Kunkle et al., 2019). T-cell activation, proliferation, and inflammatory cytokine production in response to exposure to Aβ is increased with aging and is increased further in patients with AD (Togo et al., 2002; Monsonego et al., 2003). Additionally, 30% of patients whose T cells proliferate in response to Aβ were found to have the HLA-DRB1*1501 allele which utilized Aβ_29–42_ as an epitope. More recently, it has been shown that B cells, hematopoietic cells that make antibodies, also present antigens, and express neurodegeneration-associated proteins (Nataf et al., 2019). Further, B cells expressing HLA-DRB1*1501 allele bind to peptides derived from microtubule-associated protein tau, and presenilin. While this function may contribute to tolerance to these proteins at a young age, increased inflammation and T-cell activation with age may lead to an immunogenic response to these proteins.

## Immune activation and neuroinflammation

While it is not yet known if inflammation is a primary driver in neurodegenerative diseases or if neuroinflammation is triggered by a central or systemic process, it is clear that inflammation has a crucial role in the pathogenic mechanisms of many neurodegenerative diseases, including AD, ALS, and MS (Amor et al., 2014). Microglia, the resident CNS immune cells, survey the microenvironment and respond to disease or injury. Once activated, microglia have the dual role of becoming pro-inflammatory (M1) or anti-inflammatory (M2; Guo et al., 2022). In neurodegeneration, the balance between microglia’s activation becomes shifted toward their classically activated, pro-inflammatory phenotype, producing pro-inflammatory chemokines/cytokines, such as tumor necrosis factor-alpha (TNF-α), interleukin (IL)-6, IL-1β, IL-12 (Colonna and Butovsky, 2017). Further, while T cells have long been recognized as a major player in MS, recent evidence suggested that T cells participate in the etiology of AD and ALS. T cells are the central players in the adaptive immune system, responsible for clonal expansion, differentiation, production of cytokines, and cytolytic processes. There are a number of T-cell subsets with distinct functions and roles. For the sake of discussion here, we will focus on two classes of T cells involved in neurodegenerative signals, CD4+ cells that oversee the generative of adaptive cellular immune responses and can regulate pro-inflammatory and anti-inflammatory responses and CD8+ cells which are effectors that kill antigen-bearing cells.

### Inflammatory cascade in AD development

Chronic inflammation has long been implicated in the age of onset and degree of progression of AD. Endothelial cells, a component of the blood–brain barrier, are integral as they are exposed to attack from peripheral and central inflammation leading to chronic neuroinflammation (Ludewig et al., 2019). The role of endothelial cells in establishing and sustaining this pro-inflammatory environment is an area of active inquiry (Sun et al., 2020). This may occur, in part, through polymorphonuclear and mononuclear cell adherence, diapedesis, and activation of bystander cells. Clinical evidence supporting this observation is that CD11b integrin (involved in cell adhesion and diapedesis) was upregulated in neutrophils of patients with AD and this increase was positively correlated with disease severity (Scali et al., 2002). Endothelial dysfunctions due to inflammation have been correlated to Aβ-mediated cytotoxicity, deficits in Aβ clearance, the weakening of BBB repair mechanisms, aberrant immune cell recruitment, and a direct vascular contribution to the pro-inflammatory state in vulnerable brain regions (Stanimirovic and Satoh, 2006; Pober and Sessa, 2007; Govindpani et al., 2019).

Microvascular dysfunction due to chronic inflammation leads to increased BBB permeability and infiltration of neurotoxic molecules and inflammatory factors like interleukins (ILs), interferons, MMPs, and TNFα which contribute to demyelination, axonal loss, oligodendrocyte degeneration, and cell death (Cipollini et al., 2019).

Following mononuclear cell infiltration, microglial activation and astrocytic changes occur, ultimately resulting in neuronal death. Microglial cell dysregulation as a key pathological feature of AD, by showing marked activation occurring in preclinical perfusion deficits, and resulting in metabolic dysfunction and a sustained feedback loop involving neuronal death in the affected brain areas (Dzamba et al., 2016). The resulting increase of microglial-induced cyto- and chemo-kines have been associated with astrocytic changes such as the expression of chemokine monocyte chemoattractant protein (MCP-1/CCL2), whose main function is further recruitment of leukocytes at sites of inflammation and reduction in the BBB integrity (Stamatovic et al., 2005). Cytokines released by activated microglia can modify tight junction assembly, further enhancing BBB permeability (Luissint et al., 2012) and allowing for mononuclear cell extravasation and diapedesis (Abbott et al., 2010). As part of this mononuclear cell invasion into the brain parenchyma, T cells, part of the adaptive immune response, can enter the CNS. T cells are an integral component to the immune response and monitor the CNS for infection or injury, but if left unchecked can exacerbate inflammatory damage. An association between increased T-cell infiltration in the brain and substantial amyloid deposition has been seen (McManus et al., 2014). These findings are in line with other studies that have found an increase in CD8+ T cells in the hippocampus of AD patients (Togo et al., 2002) with enhanced inflammatory activity and increased expression of cytotoxic genes (Tu and Rao, 2016). Chronic activation of the adaptive immune response could be the final player in creating sustained inflammatory feedback in AD (Whittington et al., 2017).

Given this, confirmation of cell type participation in this overarching inflammatory cascade by exploring cell signaling changes by each could prospectively contribute to the identification of a key cell type/s for therapeutic intervention.

### Inflammatory cascade in ALS development

Even with the heterogeneity in ALS pathology, neuroinflammation is a hallmark that is conserved regardless of the clinical classification. The idea of ALS being associated with an autoimmune feature was first introduced in 1986, when Appel et al. found that 19% of ALS patients had either past or present thyroid disease documented within their medical history (Appel et al., 1986). The role that the immune system plays in contributing to ALS pathology has since been investigated, suggesting alterations in microglia and astrocyte that are typically seen in ALS are mediated by genes that are associated with both cellular metabolism and immune activation, including the adaptive immune response (Rodrigues Lima-Junior et al., 2021). Although the exact relationship between immune activation and ALS pathology is unknown, evidence is mounting toward disease progression being linked to the immune function.

Within ALS, microglia undergo a phenotypic change; as the disease progresses, microglial activation and proliferation can be seen early on in the disease course (Moisse and Strong, 2006). These changes can be broadly seen in regions with only mild motor neuron loss, within the motor cortex, motor nuclei of the brainstem, the corticospinal tract, and the ventral horns of the spinal cord (Henkel et al., 2009). Microglia express higher levels of their M2 markers, which are neuroprotective toward motor neurons, at early stages of the disease (Liao et al., 2012). However, there is a shift toward their neurotoxic, pro-inflammatory M1 phenotype during disease progression which may contribute to neuronal death (Zhao et al., 2010; Tang and Le, 2016). It is likely this shift is responsible for exacerbating pathology, accelerating neuronal death and disease progression. Motor neuron death can be a direct result from microglial activation *via* their production of pro-inflammatory cytokines, by microglia-mediated protein oxidative pathology (Krieger et al., 1992; Almer et al., 2001; Shibata et al., 2001), by TNF-α-mediated apoptotic mechanisms, and by NO-induced apoptotic pathways (He et al., 2002; Raoul et al., 2002). In addition to an increased number of microglia, TNF-α is also increased before the appearance of motor deficits in SOD1 mutant mice and rats (Moisse and Strong, 2006). These preclinical findings are also supported clinically, using imaging. Using (11C)(R)-PK11195 positron emission tomography, microglial activation was shown to be increased with disease severity, suggesting microglial activation is an early feature, not only at disease end stage (Turner et al., 2004). While the inflammatory products released by activated microglia can directly cause motor neuron injury/death, the mechanism(s) leading to the induction of the microglial response remains unknown.

In addition to the changes seen within the innate immune system, changes within the adaptive immunity have also been linked to ALS pathology. Several studies have reported T-cell infiltration within the brain and spinal cords of ALS patients and murine models, with morphological and functional abnormalities within the T-cell subsets (Troost et al., 1990; Engelhardt et al., 1993; Mantovani et al., 2009). During the symptomatic stage, SOD1^G93A^ mice have an increase in both CD4+ and CD8+ T cells that infiltrate the CNS, particularly within the spinal cords (Beers et al., 2008; Chiu et al., 2008). Investigators demonstrated an increased number of CD44+/CD62L– T cells within the CNS, with the majority of the infiltrating CD8+ T cells being effector memory T cells as well as an increase in the number of CD25+ CD8+ T cells as pathology progresses (Coque et al., 2019). These processes likely lead to accelerate symptomatic progression rather than initiate the disease as suggested by studies of SOD1 mice depleted of CD8 cells.

The accessory role of the innate and adaptive immune system is also confirmed clinically. A longitudinal analysis of ALS patients showed an increased number of neutrophils and monocytes negatively correlated with the ALSFRS-R score, but not with the rate of disease progression. Further, increased levels of NK and memory TH2 T cells were correlated with a lower risk of death; however, increased CD4+/CD45R+ effector memory and CD8+ T cells were correlated with a higher risk of death (Cui et al., 2022). It is proposed that circulating immune cells enter the CNS through a disrupted blood–brain barrier (BBB). Once inside the CNS, these peripheral immune cells affect disease progression by secreting pro-inflammatory cytokines and by influencing other cells (Butovsky et al., 2012; Murdock et al., 2015; Zamudio et al., 2020). The data highlight the importance of the immune systemin ALS pathology and disease progression.

### Inflammatory cascade in MS development

Multiple sclerosis is an autoimmune disease, and in both experimental autoimmune encephalitis (EAE) and MS inflammatory demyelination is believed to be mediated by activated CD4+ T cells of a pro-inflammatory (Th1 and Th17) phenotypes that secrete interferon gamma, IL-6, and IL-17 and have the capacity to cross the BBB (Wekerle et al., 1986; Hickey et al., 1991; Flugel et al., 2001; Høglund and Maghazachi, 2014). Th17 cells may be involved in breaking down the integrity of the BBB through secretion of IL-17 and IL-22 (Tzartos et al., 2008). Further, CD8+ T cells that are observed in fresh MS plaques contribute to autoimmune encephalomyelitis. However, while MS is considered primarily a T-cell-mediated autoimmune disease, the underlying pathophysiology of MS is much more complex. T-cell reactivation after initiation of the immune response in the periphery is amplified through recognition of self-antigens presented by resident antigen-presenting cells and in response to cytokines secreted by these cells. Antigen presentation in the CNS requires the induction of MHC/HLA class II molecules (upregulated upon glial activation) as well as the upregulation of adhesion and co-stimulatory molecules on microglia. Inflammatory T cells from the periphery that have translocated into the perivascular space recruit additional T cells as well as B cells, NK cells, dendritic cells, and microglia to the site of immune activation (Zajicek et al., 1992).

These cells secrete cytokines that in turn contribute to tissue damage. B cells, plasma cells, and antibodies also are commonly found in active CNS lesions in patients with MS and in the blood of patients with chronic inflammatory demyelinating polyradiculoneuropathy. B cells and their related plasma cells isolated from CNS lesions as well as from the cerebrospinal fluid (CSF) show signs of clonal expansion and hypermutation and may contribute to the development of oligoclonal antibodies, a diagnostic hallmark, produced within the CSF of MS patients. Within the CNS, antibody deposition is associated with complement activation and demyelination.

There are four immunopatterns observed in CNS early active lesions of MS patients (Lucchinetti et al., 2000; Popescu et al., 2013; Metz et al., 2014). The early active lesions were defined by oligodendrocyte destruction and myelin protein loss, evidence of complement activation, and patterns of demyelination. Patterns I and II involve T-cell-mediated autoimmunity without (Pattern I; 15% of biopsies) and with (Pattern II; 58% of biopsies) B-cell involvement. Patterns III (26% of biopsies) and IV (1% of biopsies) show primarily oligodendrocyte loss and may indicate a viral or toxin-based mechanism. From analysis of the CSF of MS patients, oligoclonal bands are predominantly found in patients with Pattern I lesions whereas CSL/serum ratio (a measure of BBB integrity) was increased in patients with Pattern II and III lesions (Jarius et al., 2017). Interestingly, although there was heterogeneity in the lesion type among MS patients, the immunopattern of active plaques stayed consistently homogenous within a patient. In chronic MS, white-matter lesions show a more homogenous pattern with regard to underlying pathophysiological events, but remain heterogeneous with regard to degree of inflammation, demyelination, and axonal loss (Breij et al., 2008; Dziedzic et al., 2010; Frischer et al., 2015; Luchetti et al., 2018).

Because of this understanding of the pathophysiology underlying the development of CNS lesions in MS, several therapies focused on immune modulation have been developed as effective therapies for this debilitating disease.

## Bioenergetic changes in each disease and changes during disease progression

Bioenergetics and mitochondrial function are essential for generating and maintaining proper energy levels through the production and utilization of ATP. In addition to their role in producing ATP, mitochondria also play an important role in phospholipid biogenesis, calcium homeostasis, and apoptosis (Smith et al., 2019). Under normal conditions, glucose is the primary fuel for the brain, which uses about 16% of the total oxygen consumed for aerobic oxidation of glucose to carbon dioxide and water (Costantini et al., 2008).

### Bioenergetic changes in AD pathology

In AD, cognitive decline correlates with decreased cerebral glucose metabolism, and found that hypometabolism was a better indicator of cognitive dysfunction than structural changes, such as hippocampal atrophy (Sigurdsson et al., 2017). This is, in part, due to the dependence of the central nervous system on adequate energy to maintain the demands of neuronal function. This puts the brain at risk for declines in cognitive function if the supply of glucose or oxygen or both is interrupted. In line with other studies and the dominating hypothesis of the pathological development of AD, this decreased blood flow—impaired glucose metabolism as well as impaired cell waste removal—can result in insoluble Aβ fragments and neurofibrillary tangles (NFTs) and contribute to another prominent feature that has been recognized to play a factor in aging and in the progression of multiple neurodegenerative diseases including AD: oxidative stress.

Increased production of ROS is associated with age- and disease-dependent loss of mitochondrial function, altered metal homeostasis, and reduced antioxidant defenses, which directly affect synaptic activity and neurotransmission in neurons, leading to cognitive dysfunction (Tönnies and Trushina, 2017). This is primarily *via* the accumulation and marked concentration increases of reactive oxygen species (ROS)—further producing neuronal damage, cognitive decline, vascular dysfunction and, of course, a sustained deposition of cerebral Aβ (Faraci, 2006). This has been observed in APP over-expressing transgenic mice, in which antioxidant therapy or over-expression of free radical scavenger superoxide dismutase in these models reverses Aβ-mediated cerebrovascular dysfunction (Park et al., 2004).

This increased production of ROS has been observed to be linked to mitochondrial dysfunction. Specifically, disruption in glucose metabolism associated with early mitochondrial dysfunction was detected in multiple animal models and AD patients not only may be a direct determinant of oxidative stress and synaptic dysfunction, but that they contribute to early disease mechanisms before any evidence of Aβ or tau pathology (Trushina et al., 2012). Ultimately, this means AD produces several bioenergetic changes which alters cerebral blood flow regulation, reduces the brain’s redox buffering capacity, and depletes vascular metabolic reserves (Flannery and Trushina, 2019).

Such bioenergetic changes include changes in glucose transport and mitochondrial hypometabolism. The glucose transporter, GLUT1, mediates glucose transport at the BBB into the brain and has been well established to undergo significant reductions in AD (Winkler et al., 2015). Further, in a model of vascular dementia, GLUT1 was reduced in both the hippocampus and the cortex preceding Aβ deposition and persisting well after (Carnevale et al., 2012). Alternatively, insulin is known to play several roles within the CNS including regulation of neuronal survival, feeding behavior, cognition, and most importantly regulation of tissue metabolism by controlling cellular glucose uptake—accessing CNS regions by crossing the BBB *via* insulin receptor (InsR)-mediated transport, and has been shown to be altered in post-mortem brain tissue from AD patients (Griffith et al., 2018). Meanwhile, due to an increase in studies providing evidence of a causal role of brain hypometabolism and mitochondrial bioenergetic deficits (Yao et al., 2011), a subsequent increase in studies is seeking to explain how mitochondrial complex I (NADH:ubiquinone oxidoreductase—the first and the largest complex of the electron transport chain (ETC)) and complex IV (cytochrome c oxidase – the last enzyme in the respiratory ETC) offered the most profound reduction in levels of Aβ, phosphor-tau, and cognitive decline in three animal models of familial AD (Moreira et al., 2010; Zhang et al., 2015).

Linking OS induction—primarily due to mitochondrial dysfunction—could possibly serve as a springboard for studies examining the subsequent intra- and inter-cellular cascades that are activated and their role in the progression of AD pathology. We hope that this review will add to the body of evidence aimed at solidifying vascular oxidative stress and its induction of elevated Aβ as an emerging key pathogenic factor in AD (Simpson et al., 2010).

### Bioenergetic changes in ALS pathology

Within ALS, mitochondrial dysfunction has been observed within various cell types and tissues that are implicated with contributing to disease pathology. Mitochondrial abnormalities have been seen within motor neurons, microglia, and astrocytes in both *in vivo* and *in vitro* models (Obrador et al., 2021). These changes result in mitochondrial having decreased ATP production, loss of calcium homeostasis, increased pro-apoptotic signaling, and have impaired axonal transport (Smith et al., 2019). In addition, the four most prevalent genes associated with ALS (SOD1, C9orf72, FUS, and TDP43) all have been linked to mitochondrial function, highlighting the importance of proper mitochondrial function in ALS (Abel et al., 2012; Mejzini et al., 2019).

Bioenergetic alterations are one of the first observed early changes seen in the pathophysiology. In presymptomatic SOD1^G93A^ mice, motor neurons demonstrate lower respiration, with a reduction in both total cellular and the mitochondrial steady-state ATP levels (Szelechowski et al., 2018). Clinically speaking, patients with sporadic ALS also have reductions in cellular respiration (Borthwick et al., 1999; Wiedemann et al., 2002), implying that bioenergetic changes are a potential driving force in ALS pathology as a whole, not just in those with mutations in genes associated with mitochondrial function. In both sporadic and familial ALS motor neurons, hypo-oxidation and hyper-glycolysis can be seen. Hyper-glycolysis within the motor neurons of ALS patients is likely a result of a compensatory mechanism designed to overcome the reduced ATP being generated through oxidative phosphorylation (Hor et al., 2021). Additionally, bioenergetic analysis of mitochondrial respiration from iMNs from both sporadic and familial ALS patients reliably had lower basal oxygen consumption rates (OCR) compared to controls, consistent with lower mitochondrial ATP production through oxidative phosphorylation. This group also showed ATP production through glycolysis was found to be lower in every ALS cell line (Singh et al., 2021).

In addition to the direct link mitochondrial dysfunction plays in neurotoxicity, improper mitochondrial function can lead to many indirect consequences resulting in cellular toxicity. These can lead to downstream effects like excitotoxicity through glutamate neurotransmitter release (Reyes and Parpura, 2008), improper axonal transport (Mandal and Drerup, 2019), and the accumulation of ROS leading to increased oxidative stress (Barber and Shaw, 2010). Although the mechanisms leading to ALS remain elusive, mitochondrial dysfunction, glucose hypometabolism, impaired metabolic support from glia, and aberrant energy signaling all create energy imbalances seen in both ALS patients and model systems (Nelson and Trotti, 2022). Despite the heterogeneity seen in ALS patients, the direct and indirect effects of impaired mitochondrial function remain conserved, strongly implying mitochondrial dysfunction to be a driving force in ALS pathology.

### Bioenergetic changes in MS pathology

In EAE, mitochondrial function and energy production is reduced and this reduction precedes the onset of neurological dysfunction (Sadeghian et al., 2016). The loss of optimal mitochondrial function and the resulting energy deficit leads to impairment of impulse transmission, axonal transport, and neuronal ion trafficking (Adiele and Adiele, 2019; Correale et al., 2019). Activation of the inflammatory system, including microglia and macrophages, further exacerbates mitochondrial dysfunction as disease progresses (Kalman and Leist, 2003; Witte et al., 2014).

Ultrastructural analysis of spinal cord lesions in MS shows depletion of mitochondrial content and increased axonal swelling (Mao and Reddy, 2010). Demyelinated axons are more susceptible to reduction in bioenergetics than myelinated axons supporting the hypothesis that mitochondrial dysfunction contributes to MS progression (Mahad et al., 2008; Fischer et al., 2012). Additionally, bioenergetic insufficiency has remarkable impacts on oligodendrocyte precursor cells, the cells that support remyelination in the CNS are sensitive bioenergetics deficits. The inability of OPC proliferate and differentiation secondary to mitochondrial dysfunction leads to loss of neurological function (Kuhlmann et al., 2008; Ziabreva et al., 2010; Kremer et al., 2011). In Pattern III lesions, which have the hallmark of loss of oligodendrocytes, there is a reduction in mitochondrial density and the level of Complex IV of the electron transport chain (Mahad et al., 2008). Mitochondrial loss and reduced mitochondrial gene expression is observed in neurons of MS tissues (Dutta et al., 2006). In neurons, mitochondrial deficits are associated with reduced activity of complexes I, III, and IV of the electron transport chain and axonal degeneration. Mitochondrial dysfunction and the resulting bioenergetics crisis is a key driver of neurodegeneration in MS (Lassmann and van Horssen, 2011).

Oxidative stress (OS), an outcome of mitochondrial dysfunction, is an important pathophysiological process in the development of MS (Lu et al., 2000; Aboul-Enein and Lassmann, 2005; Glass et al., 2010; Campbell et al., 2011; Fischer et al., 2012). The CNS is particularly susceptible to OS due to the high metabolic rate and the high level of polyunsaturated fatty acids in the membranes of CNS cells (Patergnani et al., 2017; Barcelos et al., 2019). In a study of MS patients, increased levels of markers of OS (thiobarbituric acid reacting substances and oxidized proteins) were seen in the plasma and saliva (Karlík et al., 2015). Oxidative damage to mitochondria precedes the infiltration of inflammatory cells into the CNS in EAE (Qi et al., 2006). Reactive oxygen species (ROS) play key role in many processes underlying MS pathogenesis (van Horssen et al., 2008, 2011; Lassmann and van Horssen, 2016). Activated microglia and macrophages, a major component of MS lesions, generate high amounts of ROS by myeloperoxidase, xanthine oxidase, and NADPH oxidases activities (Gray et al., 2008a,b). ROS interact with nitric oxide to generate the highly reactive peroxynitrite in MS resulting in the peroxidation of macromolecules in oligodendrocytes, astrocytes, macrophages, and damage axons (Cross et al., 1998; Liu et al., 2001; Qin et al., 2007; Haider et al., 2011). As described above for AD, OS contributes to the disruption of the BBB and enhances leukocyte migration and function into the CNS. Extensive oxidative damage is seen in active demyelinating lesions, particularly in reactive, hypertrophic astrocytes, and macrophages that have phagocytosed myelin. Among the neuroglial cells, oligodendrocytes, the cell that generated the myelin sheath, are more susceptible to oxidative damage than astrocytes. In part, this is due to the high metabolic demand for the production of myelin proteins, particularly during periods of active myelination and remyelination (McTigue and Tripathi, 2008).

## Targeted approach

**Figure 1:**
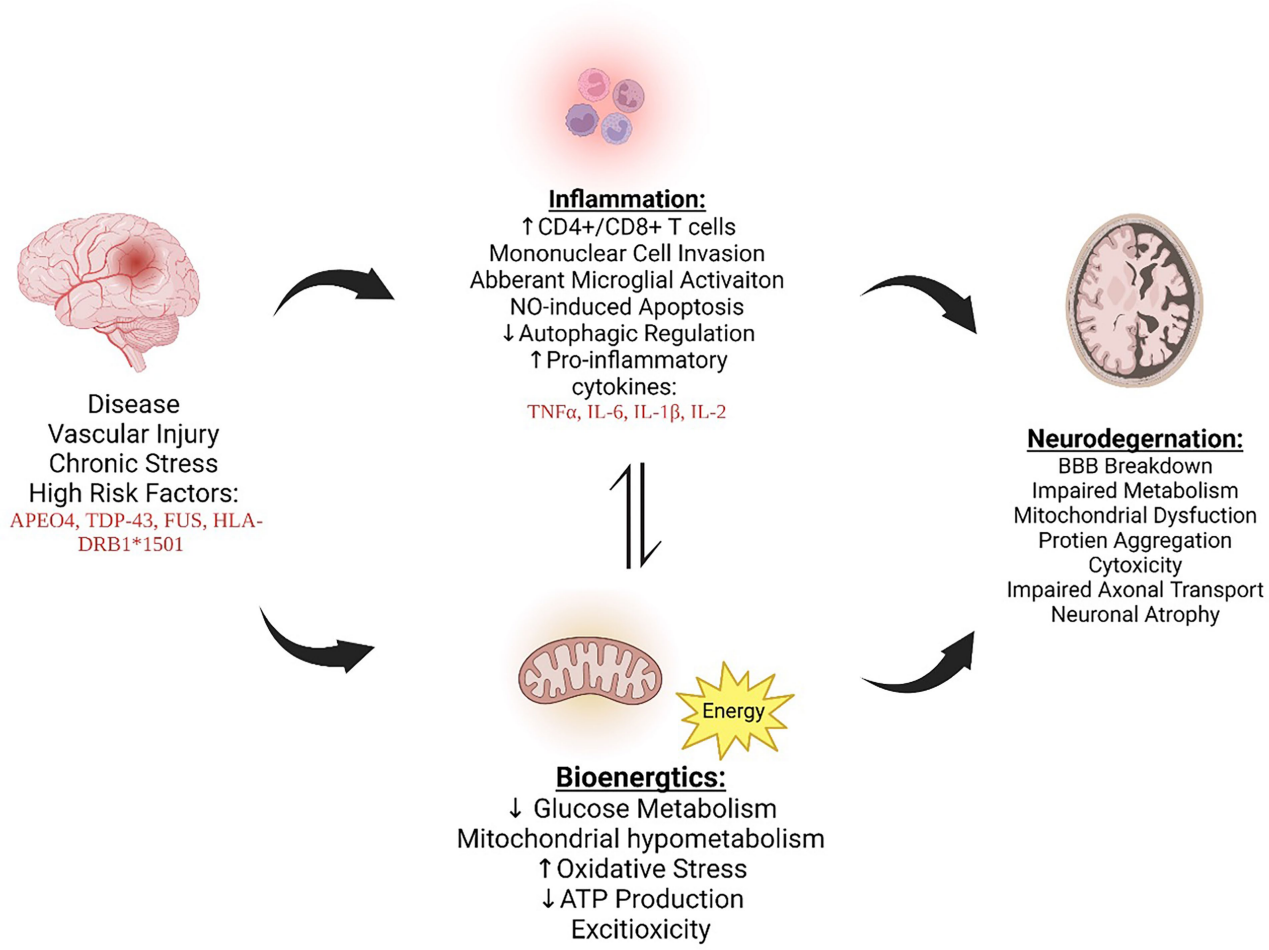
AD, MS, and ALS exhibit increased inflammation and bioenergetic changes, ultimately leading to neurodegeneration and furthering disease progression. The cross talk between inflammation and bioenergic dysfunction is dynamic and influences one another, promoting dysregulation. *Figure created with BioRender.com*.

As previously stated, while there is some overlap, AD, ALS and MS have disease-specific genes associated with an increase in prevalence. These differences provide an unparalleled challenge for developing blanket therapeutics that have a universal benefit to all NDD patients. However, focusing on the downstream mechanisms could be the future of drug development to treat all neurodegenerative diseases. Autophagy, a cellular process that is impaired and contributes to chronic immune activation and reduced mitochondrial bioenergetics, is one cellular mechanism that is implicated in all three, making it a viable target for drug development (Figure 1).

Autophagy is a vital metabolic process responsible for a wide array cellular processes and maintaining homeostasis. The cross talk between the immune system and autophagy is crucial for cell survival and maintaining the microenvironment. Autophagy plays an important role in both the adaptive and innate immune systems, contributing in pathogen removal, cytokine production, and lymphocyte survival (Misrielal et al., 2020). Ultimately, autophagy dysregulation can have consequences that ultimately lead to neuroinflammation and neurodegeneration. The relationship between autophagy and inflammation is complex, being able to both promote and downregulate each other through different mechanisms (Levine et al., 2011; Deretic et al., 2013; Liang and Le, 2015). Therefore, it is unsurprising autophagy has been pathologically linked to multiple neuroinflammatory diseases, such as AD, ALS, and MS (Muller et al., 2017; Yin et al., 2018).

Autophagy has been shown to influence the immune system through the NF-kB signaling pathway and by enhancement of APCs (Chemali et al., 2011). The NF-kB transcription factors regulate a variety of genes responsible for cell proliferation, cell survival, inflammation, and immune responses (Hayden and Ghosh, 2011). The cross talk between NF-kB and autophagy can be observed in immune cells with varying responses depending on the tissue-specific macrophages involved (Zhong et al., 2016). These cell-specific responses indicate a dual pathogenic or therapeutic role autophagy plays, depending on the external signals. In addition to NF-kB signaling, autophagy allows for APCs to properly digest pathogens and enhance MHC-I and MHC-II presentation (Chemali et al., 2011). Impaired autophagy therefore can lead to immune dysfunction and reduced clearance of cytotoxic agents.

The catalytic protein, mTOR, is a primary regulator for cellular metabolism, having a critical role in regulating autophagy. mTOR works through two main complexes, mTOR complex 1 (mTORC1) and mTOR complex 2 (mTORC2). These protein kinase complexes bind to accessory proteins to regulate cellular metabolism and promote cell growth (Kim and Guan, 2015). mTORC1 inhibits autophagy and lysosome biogenesis, impairing the ability to breakdown and remove damaged organelles and macromolecules which further adds to cellular stress. In AD, ALS, and MS, mTOR activity is increased, leading to inflammation and neuronal damage (Oddo, 2012; Li et al., 2020; Granatiero et al., 2021). Due to this phenomenon, researchers have begun to look at a pharmacological agent that attenuates mTOR activity, such as Rapamycin, to enhance autophagy. In ALS, Rapamycin administration improved symptoms in a *Drosophila* model of ALS-TDP, reducing both neuronal loss and TDP43 inclusions. Rapamycin was also shown to increase Treg levels, a characteristic associated with a slower progression of ALS (Mandrioli et al., 2018). mTOR is also implicated in cognitive decline during normal aging, and Rapamycin administration has been able to preserve brain function and mitigate cognitive decline in AD by increasing autophagy and reducing amyloid-beta and tau pathogenic proteins (Kaeberlein and Galvan, 2019; Mueed et al., 2019). Rapamycin administration is also beneficial in MS, by reducing relapsing–remitting EAE, likely by the immunosuppressive effect in the inflammatory-mediated demyelination, indicating a potential role in MS (Liang and Le, 2015).

## Conclusion

As can be deduced, the use of appropriate models is paramount in recapitulating the drivers in pathology discussed. Making the “right” choice is arguably the most important factor in gauging success of research endeavors (Sprott, 1999). A good model must not only mimic pathology, but also the complex temporal relationships that occur in neuroendocrine and immune systems and the organism (Swearengen, 2018). This allows relevant, translatable scientific data from which to draw meaningful therapeutic perspectives. The most commonly used model for AD are transgenic mice that overexpress human gene associated with familial AD, including APP, PSEN1, and APOE4 (Drummond and Wisniewski, 2017; Jankowsky and Zheng, 2017). Those for ALS include SOD1 and TDP43 mutant mice (Philips and Rothstein, 2015). And for MS, involving the EAE, TMEV infection model, and toxin-induced demyelination (Procaccini et al., 2015). Unfortunately, success rates of drugs in clinical trials of these diseases after testing in animal models have all been disappointing (Petrov et al., 2017; Hart et al., 2021). Understanding the shared pathology of these diseases may require using a shared approach to animal model selection: genetic models with high-risk factors. Genes and the environment play an intertwined role in disease onset and progression (Lovely et al., 2017). Therefore, animal models should incorporate high-risk environmental effects in studies. High-risk factors in AD include hypertension and diabetes, for ALS include smoking and hypermetabolism disorders, and for MS include smoking and obesity (Silva et al., 2019; Tsai et al., 2019; Taan et al., 2021). By combining genetic and risk factor into an animal model, conclusions drawn may provide greater transability in clinical trial than generated with a genetic model alone.

As this review highlights, neurodegenerative disorders have common underlying mechanisms which manifest in unique disease pathology depending upon risk factors, predominance of the mechanism in the disease progression, and area of the central nervous system affected. Future work should be geared toward filling the gaps in understanding the level of impact and influence these factors play in pathology development., which could lead to the development of better tools to measure disease progression. However, these commonalities, evaluated at differing levels in each disease, can at least allow a starting point for potential therapeutic interventions that can intersect through modification of these shared underlying processes. Understanding the shared aspects will allow for universal treatments that can encompass all neurodegenerative diseases.

## Author contributions

All authors listed have made a substantial, direct, and intellectual contribution to the work and approved it for publication.

## Funding

This work was supported by T32-AG061897, U01-AG063768, and P01-AG026572.

## Conflict of interest

The authors declare that the research was conducted in the absence of any commercial or financial relationships that could be construed as a potential conflict of interest.

## Publisher’s note

All claims expressed in this article are solely those of the authors and do not necessarily represent those of their affiliated organizations, or those of the publisher, the editors and the reviewers. Any product that may be evaluated in this article, or claim that may be made by its manufacturer, is not guaranteed or endorsed by the publisher.
